# Identification and characterization of compounds from *Chrysosporium multifidum*, a fungus with moderate antimicrobial activity isolated from *Hermetia illucens* gut microbiota

**DOI:** 10.1371/journal.pone.0218837

**Published:** 2019-12-20

**Authors:** Yesenia Correa, Billy Cabanillas, Valérie Jullian, Daniela Álvarez, Denis Castillo, Cédric Dufloer, Beatriz Bustamante, Elisa Roncal, Edgar Neyra, Patricia Sheen, Michel Sauvain

**Affiliations:** 1 Laboratorios de Investigación y Desarrollo, Universidad Peruana Cayetano Heredia, Lima, Peru; 2 Unité Mixte de Recherche 152 Pharmacochimie et Biologie pour le Développement, Institut de Recherche pour le Développement, Université Toulouse III–Paul Sabatier, Toulouse, France; 3 Clinical Mycology Laboratory, Instituto de Medicina Tropical Alexander von Humboldt, Universidad Peruana Cayetano Heredia, Lima, Peru; University of British Columbia, CANADA

## Abstract

The gut microbiota of insects is composed of a wide range of microorganisms which produce bioactive compounds that protect their host from pathogenic attack. In the present study, we isolate and identify the fungus *Chrysosporium multifidum* from the gut of *Hermetia illucens* larvae. Extract from *C*. *multifidum* culture broth supernatant showed moderate activity against a strain of methicillin-resistant *Staphylococcus aureus* (MRSA). Bioguided isolation of the extract resulted in the characterization of six α-pyrone derivatives (**1**–**6**) and one diketopiperazine (**7**). Of these compounds, 5,6-dihydro-4-methoxy-6-(1-oxopentyl)-2H-pyran-2-one (**4**) showed the greatest activity (IC_50_ = 11.4 ± 0.7 μg/mL and MIC = 62.5 μg/mL) against MRSA.

## Introduction

*Hermetia illucens*, or the black soldier fly (BSF), is an insect native to the Americas whose larvae quickly colonize decomposing matter. Unaffected by their pathogen-rich diet, BSF efficiently produces a range of substances relevant to the animal feed and biodiesel industries [1–2]. This suggests that BSF larvae have a potent immune system capable of defeating a wide range of pathogens present in their environment [3–5]. Furthermore, the gut of BSF larvae may also harbor beneficial microbes and fungi that also control pathogens. Therefore, BSF and the microbes and fungi associated with it are good targets in the search for new types of antimicrobial compounds relevant for human and animal medicine. Recently, a study discussed the diversity of fungi isolated from the gut of BSF larvae. Among the fungi characterized, *Trichosporon asahii* was shown to be active on strains of *Candida glabrata* and *Candida lusitaniae* [6]. Other antibacterial substances such as peptides [7,8] and lipids [9] have also been isolated from BSF, but information is still limited. The purpose of this work is to isolate and identify fungi with antimicrobial activity from the gut of BSF larvae, as well as to isolate and identify antimicrobial compounds from the *in vitro* culture of antimicrobial fungi.

## Material and methods

### Larvae rearing

The BSF larvae used in this experiment were obtained from the breeding colony established at the Universidad Peruana Cayetano Heredia (Lima, Peru) maintained at 28 ± 1°C and 70% relative humidity. Specimens were fed fresh unsterilized chicken guano for 11 days. After this time, larvae exhibited lengths between 1.5–2 cm.

### Extraction of gut from larvae and isolation of active fungi

Samples were collected in triplicate; each collection corresponded to larvae obtained from a different breeding cycle. Ten larvae were washed with 70% ethanol/water and sterilized for 15 min with UV light. Each larva was dissected longitudinally using sterile scalpels and the entire gut was removed using sterile tweezers. Portions (0.5 cm) of the midgut from each larva were pooled and combined with 200 μL of saline solution 0.89% w/v. The pooled sample was diluted (10^−1^–10^−4^) and 10 μL was seeded on 15 mL of potato dextrose agar (PDA) (BD-Difco^®^) or 15 mL Sabouraud agar (SBA) (BD-Difco^®^) both supplemented with chloramphenicol (100 mg/L) and gentamicin (50 mg/L). To discount external contamination, two controls were prepared: one consisted of the saline diluent; the other was prepared from swabs of the larvae surface after disinfection and before dissection. Controls were treated in the same manner as pooled samples. The agar plates with sample dilutions were incubated for 21 days at 30 ± 1°C. Yeast and mold strains with different morphology were separated repeatedly and grown on the same fresh media to obtain pure colonies. A previously described method [10] with small modifications (S1 Method), was used to evaluate preliminary antimicrobial activity of fungi colonies against the pathogenic bacteria *Staphylococcus aureus* subsp. *aureus* ATCC 43300 and *Salmonella enterica* subsp. *enterica* var. Typhimurium ATCC 13311.

### Extraction of DNA from the active fungus

A 100 mg sample of mycelium, 700 μL of extraction buffer (0.1 M Tris-HCl (pH 8), 20 mM EDTA (pH 8), 1.4 M NaCl, 0.2% (v/v) 2-mercaptoethanol and 2% (w/v) CTAB), and acid-washed 150–212 μm glass beads were combined in a 2 mL tube. The mycelium was lysed using Qiagen Tissue Lyser II for 30 seconds, and an aliquot of 15 μL RNAse A (20 mg/mL) was added to the tube and mixed at 55°C and 850 rpm for 30 min. Chloroform:isoamyl alcohol (24:1, 700 μL) was added, and the mixture was centrifuged at 14,000 rpm for 10 min at room temperature. The supernatant was mixed with 50 μL of 10% (w/v) CTAB and 600 μL of chloroform and centrifuged at 14,000 rpm for 10 min. The resulting supernatant was transferred to a clean 1.5 mL tube. An equal volume of ice-cold isopropanol was added, incubated at -20°C overnight and then centrifuged at 14,000 rpm for 20 min. The pellet was rinsed twice with 1 mL of 80% ethanol (4°C) and centrifuged at 14,000 rpm for 10 min. The resulting pellet was left to dry at room temperature for 3 hours. DNA recovery was quantified using a Nanodrop spectrophotometer. The isolated DNA was separated by electrophoresis on a 1.5% agarose gel to verify integrity. The extracted DNA was stored at -20°C until use.

### PCR amplification and sequencing of the active fungus DNA

Two zones of fungal DNA were amplified by conventional PCR: ITS1-2 rRNA with the universal primers ITS1 (TCCGTAGGTGAACCTGCGGG) and ITS4 (TCCTCCGCTTATTGATGGC); and the D1/D2 domains of large sub-unit (LSU) ribosomal DNA (rDNA) with the universal primers NL1 (GCATCAATAAGCGGGAGGAAAG) and NL4 (GGTCCGTGTTTCAAGGGG). Reactions were prepared by combining 25 μL of KOD Hot start Master Mix (Sigma Aldrich^®^), 1.5 μL of each primer (10 μM), 18.5 μL of nuclease free water, and 3.5 μL DNA extract (20 ng/μL). DNA was amplified using the following conditions on a thermocycler: 94°C for 5 min, followed by 35 amplification cycles (94°C for 30 seconds, 55.7°C for ITS1-2 or 58.1°C for D1/D2 for 30 seconds, and 72°C for 60 seconds), with a final incubation of 72°C for 7 min. The PCR product was verified using 1.5% agarose gel electrophoresis and sequenced (Macrogen USA). The sequence of each PCR product was analyzed using Sequencher 5.4.6 Software (Gen Codes Corporation). Subsequently, the Nucleotide BLAST tool (NCBI) was used to align the observed sequence to known reference sequences.

### Preparation of the active fungus broth extract

A culture of the active fungus (1x10^5^ spores/mL) was used to inoculate 50 mL of Sabouraud broth (BD-Difco^®^). The inoculated broth was incubated at 30°C and 150 rpm for 2 days. The resulting culture was divided into two parts and transferred to flasks containing 500 mL of dextrose broth which was then incubated at 30°C and 150 rpm for 3 days. This operation was repeated until 10 L of culture were obtained. Mycelium was separated from the broth by vacuum filtration. The broth was extracted with one volume of ethyl acetate. The organic layers were collected and a rotavapor was used to remove the ethyl acetate solvent, resulting in 1.5 g of crude extract.

### TLC-direct bioautography—dot-blot assay

Activity of fungus broth extract was tested using a thin layer chromatography-direct bioautography (TLC-DB) dot-blot assay described by Jesionek et al. [11] with some modifications. Duplicates of 100, 200, or 300 μg of extract and 0.25 μg of tetracycline control was spotted on 8 x 3 cm aluminum-backed silica gel 60 F264 (Merck^®^) plates. Plates were added to 90 mm Petri dishes and sterilized under UV light for 15 minutes to avoid contamination. Then, 5 mL of nutritive agar (BD-Difco^®^) containing the pathogenic bacteria (1x10^6^ CFU/mL) was distributed over each plate. After solidification of the medium, the Petri dishes were incubated at 37°C for 18 hours. Bacterial growth was revealed by adding 200 μL of a 5 mg/mL solution of MTT (3-(4, 5-dimethylthiazol-2-yl)-2, 5-diphenyl tetrazolium bromide, Sigma-Aldrich^®^) in PBS 1X (pH 7.4) over the agar followed by an incubation at 37°C for 3 hours. Inhibition zones were revealed as a yellow color against a purple background (S1 Fig). The same method was used to test the activity of fractions obtained from chromatography assays.

### Compound isolation

The crude extract (1.5 g) was separated on silica gel by MPLC with a gradient of CH_2_Cl_2_–MeOH (v/v, 0:1 to 1:0) to provide 16 fractions (CM1-CM16). Fraction 5 showed the highest activity on the TLC-DB test. This fraction was separated again using silica gel and a gradient of petroleum ether-ethyl acetate (v/v, 90:10 to 80:20) resulting in 8 fractions (CM5.1 –CM5.8). Fraction CM5.3 was identified as **2** (6.4 mg), fraction CM5.5 as **4** (1.7 mg) and fraction CM5.8 as **6** (8 mg). Fraction CM5.7 was filtered on a Sephadex LH-20 column using CH_2_Cl_2_ as eluent to yield **1** (4.2 mg). Compounds **3** (16.3 mg) and **5** (4.9 mg) were obtained from the purification of fraction CM9 on silica gel using a gradient of petroleum ether-ethyl acetate (v/v, 80:20 to 60:40). A fractionation of CM10 on silica gel with a solvent system of MeOH-CH_2_Cl_2_ (v/v, 80:20 to 100:0) resulted in the isolation of **7** (5 mg).

### Compound identification

Optical rotations were measured on a JASCO P-2000 polarimeter. Mass spectra were obtained using a Thermo Scientific LTQ Orbitrap XL mass spectrometer. NMR data were obtained using a Bruker AVANCE 500 NMR spectrometer for compounds **1**, **4**, **6** and **7** and on Bruker AVANCE 300 NMR spectrometer for compounds **2**, **3** and **5**. Analysis of spectroscopic data were carried out using Xcalibur^^®^^ and MestreNova^^®^^ software. A detailed description of the spectroscopic information of each compound as well as a copy of the spectra are presented as supplementary material (S1 Data, S2 Data).

### Antibacterial activity assays

The half-maximal inhibitory concentration (IC_50_) was determined using microdilution [12,13]. Mueller Hinton Broth (MHB, 7 mL) was inoculated with the pathogenic bacteria and incubated at 37°C for 24 hours. Meanwhile, isolated compounds were dissolved in DMSO. Dilutions were prepared in 96-well plates mixing prepared DMSO solutions with MHB medium to a final volume of 50 μL. Then, an inoculated aliquot (50 μL) was added to the dilutions. Final concentrations of compounds in each well ranged from 500 to 0.98 μg/mL, bacterial density was 5 x 10^5^ CFU/mL and the final concentration of DMSO was less 1%. After 24 h of incubation at 37°C the optical density (OD) was read at 595 nm. Tetracycline was used as a positive control in a range of 0.3–0.025 μg/mL. A Probit analysis was performed to determine the IC_50_ of the compounds. The minimum inhibitory concentration (MIC) was determined as the lowest concentration that did not present turbidity or bacterial growth.

## Results and discussion

### Isolation and identification of the active fungus

**Fig 1:**
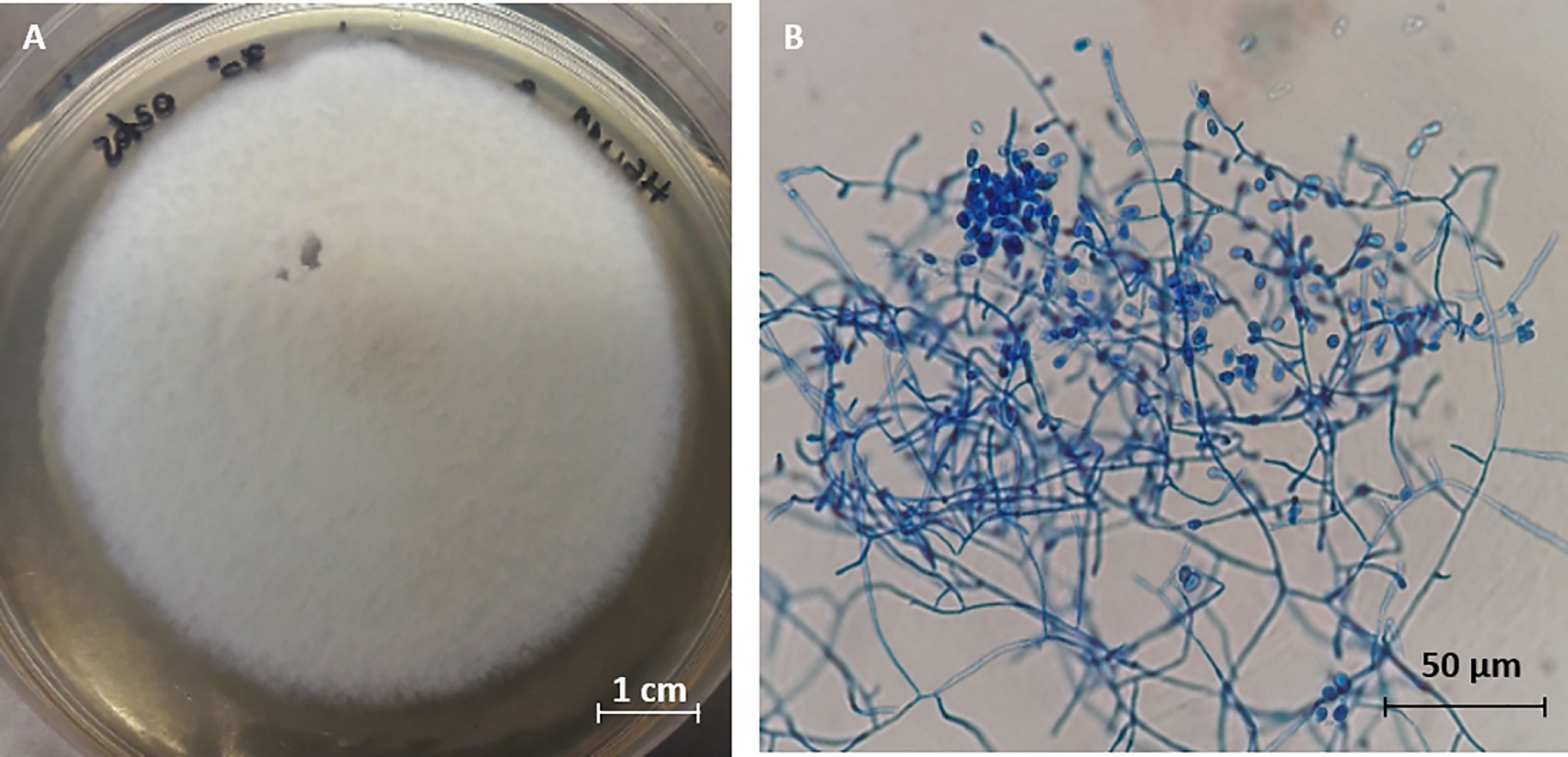
*Chrysosporium multifidum* isolated from *H*. *illucens* gut after 7 days of incubation at 30°C. Macroscopic view (A). Microscopic view of pyriform microconidia and hyaline septate hyphae (B).

Culture of solutions prepared from *H*. *illucens* gut resulted in the isolation of 25 cultivable fungal strains with different morphotypes. The most active strain (HGU11_8) was selected by submitting all isolated fungal strains to a preliminary antimicrobial test against *S*. *aureus* and *S*. Typhimurium (S1 Method, S1 Table). We then proceeded to identify this active strain by DNA sequencing. An NCBI Blast search of the resulting sequence resulted in 100% identity and coverage with GenBank accession numbers AB861747.1 and AB359438.1, which correspond to *Arthroderma multifidum*, a telomorphic ascomycete [14,15]. However, no telomorphic stage was observed in the prepared cultures. Instead, abundant pyriform microconidia and hyaline septate hyphae (Fig 1) were observed, which indicate the presence of the anamorphic form of *A*. *multifidum* named *Chrysosporium multifidum* (GenBank accession numbers: MK982149 and MK982181). The use of this fungal growth stage allowed us to determine the antimicrobial activity of its culture supernatant with different experimental methods.

This species is an opportunistic or possibly pathogenic saprotroph commonly found in soil. However, it seems to behave as an endosymbiont with *H*. *illucens*. *Chrysosporium* has been isolated from chicken guano samples [16], which makes it likely that it BSF larvae acquired it from their diet. It is also possible that this fungus was selected by the BSF’s biological system, as the fungus may provide enzymes or even beneficial antimicrobial substances to the larvae in exchange for an environment with enough nutrients for growth and development [17]. It is known that some of these selected fungi can survive in glandular cavities or cuticular invaginations called mycangia where they can develop and reproduce while being transported to new hosts by the insects [18,19].

### Isolation of chemicals from *C*. *multifidum* broth extract

**Fig 2:**
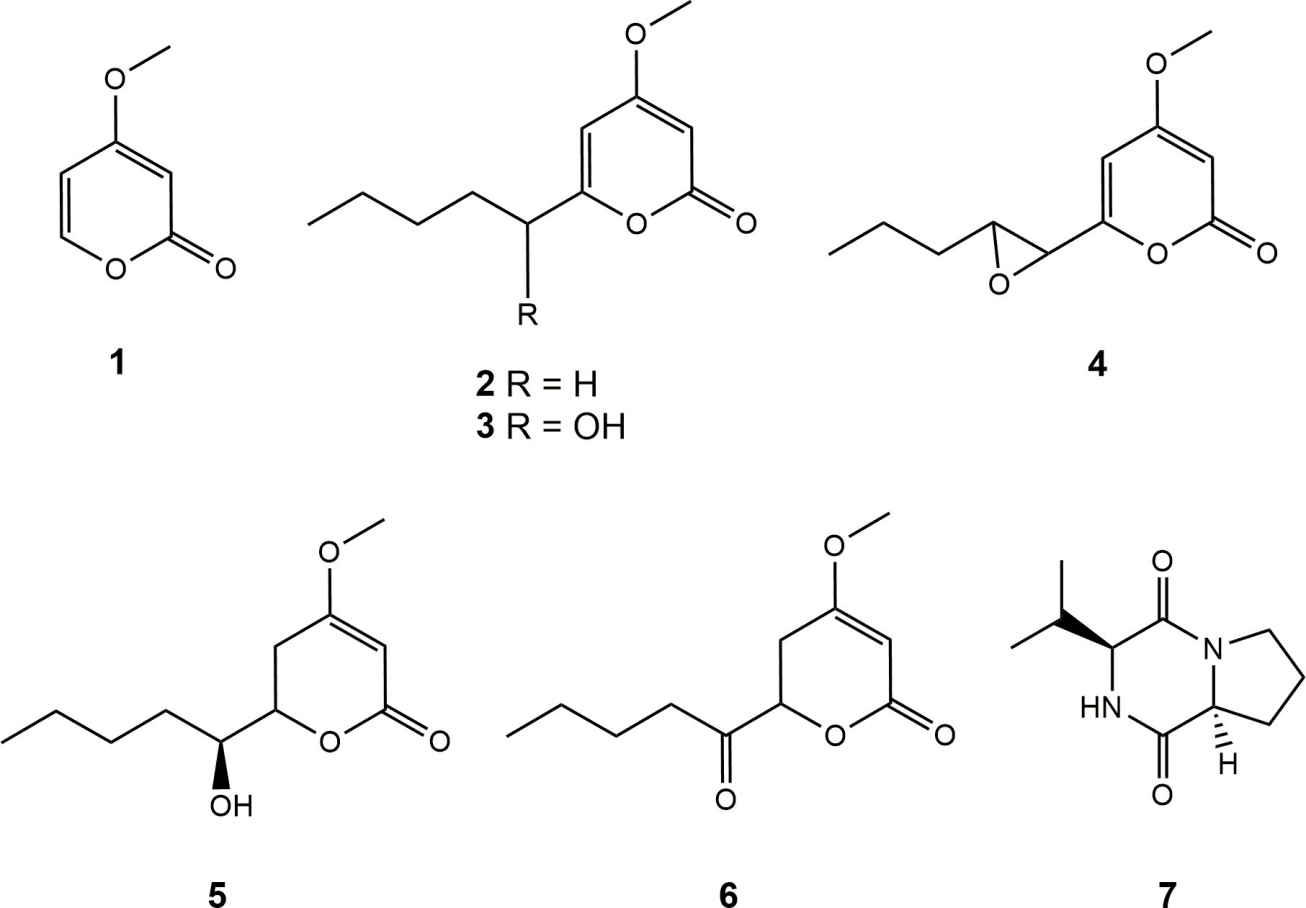
Structures of compounds of 1–7 isolated from *C*. *multifidum* broth extract.

Bioguided analysis of the ethyl acetate extract of *C*. *multifidum* broth resulted in the isolation of 4-methoxy-2H-pyran-2-one (**1**) [20], 4-methoxy-6-pentyl-2H-pyran-2-one (**2**) [21], 6-(1-hydroxypentyl)-4-methoxy-pyran-2-one (**3**) [21,22], 6-[8-propyloxiran-1-yl]-4-methoxy-pyran-2-one (**4**) [23], pestalotin (**5**) [24,25], 5,6-dihydro-4-methoxy-6-(pentanoyloxy)-2H-pyran-2-one (**6**) [22,25] and cyclo-(L-Pro-L-Val) (**7**) [26]. All the compounds (Fig 2) were identified by comparison of their spectroscopic data (HRMS and ^1^H and ^13^C NMR) with literature-reported values, as well as careful examination of their 2D NMR spectra (COSY, HSQC, HMBC). Optical rotations were also coherent with published data, except for (**4**), for which we found an optical rotation close to zero indicating the possible isolation of a racemic mixture (found [α]^20^_D_ -4.3, c = 0.16, MeOH/CH_2_Cl_2_ 9/1; published [α]^25^_D_ -98.7, c = 0.6, MeOH) [23].

This study is the first published chemical characterization of *C*. *multifidum*. The literature describes several chemical prospecting works carried out on species of the *Chrysosporium* genus, which led to the discovery of groups of compounds such as adenopeptines [27], nucleosides [28], zearalenone derivatives [29], benzoquinones [30], naphthoquinones [31], anthraquinones [32], benzolactones [33], naphthopyrones [34], naphthalenes [35], phenyl-2(1H)-pyridinones [36], alkylphenols [37], bisdechlorogeodins [38], sterols [39,40], and caryophillenes [41]. However, there is no prior record of any α-pyrone derivatives, so this would be the first report of these compounds within the genus. Compound **7** is also reported here for the first time within the *Chrysosporium* genus; but it has also been reported from cultures of other fungi and bacteria [42,43].

### Biological analyses

The α-pyrone **4** was the most active compound in the bioautography test. The antimicrobial activity of this compound was further quantified (Table 1), which indicates only moderate activity compared with the tetracycline control. Other known compounds isolated from *Chrysosporium* spp. have displayed biological activities as antitumor [36], antifungal [28,29,40,41] and cytotoxic [33] agents. However, only naphthoquinone-type compounds isolated from *C*. *queenslandicum* [31] have been shown to be active against the gram-positive bacteria *Micrococcus luteus* and *Bacillus subtilis* with MIC values close to those obtained in this work. On the other hand, both natural and synthetic α-pyrones have shown antimicrobial and antifungal activity on a variety of species [44,45]. Substitutions in positions 4 and 6 of the pyrone ring may be related to this activity. Subsequent trials should be carried out to test the activity of all derivatives of isolated α-pyrones on other groups of bacteria including gram-negative species.

**Table 1:** Antimicrobial activity of compound 4 against MRSA.

Compound	IC_50_ (μg/mL)	MIC (μg/mL)
**4**	11.4 ± 0.7	62.5
**Tetracycline**	0.1 ± 0.02	0.4

## Conclusion

A total of 25 different fungi were isolated from the gut of *H*. *illucens* larvae fed with chicken guano. These colonies were tested on methicillin-resistant *Staphylococcus aureus* (MRSA) ATCC 43300 and *Salmonella* Typhimurium ATCC 13311. The fungal specimen with the greatest activity was subsequently named as *Chrysosporium multifidum*. A broth culture of the fungus was prepared and seven compounds present in the broth were characterized using chromatographic methods and TLC-DB. The α-pyrone derivative **4** was shown to have moderate activity (MIC 62.5 μg/mL) against MRSA. These first results from the exploration of the microbiota of *H*. *illucens* indicate that it may be a useful source of antimicrobial compounds that have activity against otherwise resistant pathogens. Furthermore, this opens a window to explain how *H*. *illucens* larvae control the pathogenic microbes ingested in contaminated food with an endosymbiotic fungus.

## Supporting information

S1 Methods Preliminary evaluation of antimicrobial activity of yeast and molds. (DOCX)

S1 Fig Results of bioautography test for *C. multifidum*. (TIF)

S1 Table Results of preliminary antimicrobial test of yeast and molds isolated from *H. illucens* against *S. aureus* and *S. Typhimurium*. (XLSX)

S1 Data RMN and MS spectra of isolated compounds. (DOCX)

S2 Data Spectroscopic data of compounds isolated from the culture broth of *C. multifidum*. (DOCX)
