# Supplementary material for: Identification and characterization of compounds from Chrysosporium multifidum, a fungus with moderate antimicrobial activity isolated from Hermetia illucens gut microbiota
Source: PLoS One. 2019 Dec 20;14(12):e0218837. doi: 10.1371/journal.pone.0218837 (PMC6924690; doi:10.1371/journal.pone.0218837)
Supplement: S1 Methods — (DOCX) [file pone.0218837.s001.docx]

**S1 Methods. Preliminary evaluation of antimicrobial activity of yeast and molds**

Evaluation of yeast antimicrobial activity

Strains of yeast (1x10^6^ CFU/mL) were added to 15 ml of Sabouraud agar (ASB), poured onto Petri dishes and incubated for 24 h at 30°C. After yeast growth, 8 mm diameter slabs were cut out and put on a petri dish containing 15 ml of Nutritive Agar (NA) with pathogenic bacteria (1x10^6^ CFU/mL). Petri dishes were incubated for 24 h at 30°C. Following the incubation, the diameter of the pathogenic bacterial growth inhibition zone was measured. Tetracycline (30 µg) was used under the same conditions as a positive control. Experiments were carried out in triplicate.

Evaluation of antimicrobial activity of molds

A 10 µl aliquot of 1x10^3^ CFU/mL spore stock solution was inoculated into a 2 mm hole in the center of a Petri dish containing 24 ml of ASB. The dish was incubated for 6 days at 30°C until radial fungal growth was observed.

The pathogenic bacteria were seeded around the fungus using a sterile swab dipped in a suspension of 1x10^6^ CFU/ml. The plate was incubated for 24 h at 30°C and the diameter of the pathogenic bacteria grown inhibition zone was measured. Tetracycline (30 µg) was used under the same conditions as a positive control. Experiments were carried out in triplicate.

Data normalization

The fungal antimicrobial potential (AP) was calculated to compare the data from both methods. The AP is the ratio between the diameter of the inhibition halo and the diameter of the fungal colony (Pereira et al., 2013). Fungi with antimicrobial activity have an AP greater than 1, fungi with no activity have an AP value of 1.

To select the most active and interesting strains, the contrast of the inhibition halo’s borders was included as an additional parameter. It was assigned a signal with (+++) indicating a very sharp halo and (+) indicating a diffuse halo. The results are reported in supplementary information (S1. Table).


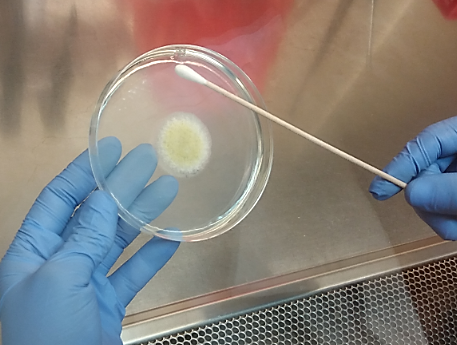

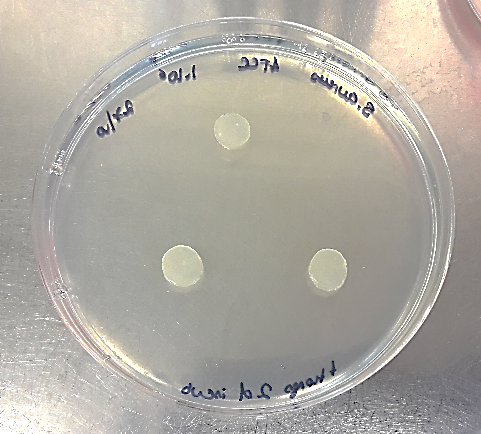


A

B

**Fig. 1: Methods for Preliminary evaluation of antimicrobial activity of yeast and molds. (**A) Yeast agar slab method, the 3 small circles are the yeast agar slabs placed on the surface of the NA with pathogenic bacteria. (B) Method for antimicrobial activity of molds. Mold is first grown in the center of the plate, later pathogenic bacteria are inoculated with a swab around the mold.
