# Supplementary figures and images for: Identification and characterization of compounds from Chrysosporium multifidum, a fungus with moderate antimicrobial activity isolated from Hermetia illucens gut microbiota
Source: PLoS One. 2019 Dec 20;14(12):e0218837. doi: 10.1371/journal.pone.0218837 (PMC6924690; doi:10.1371/journal.pone.0218837)

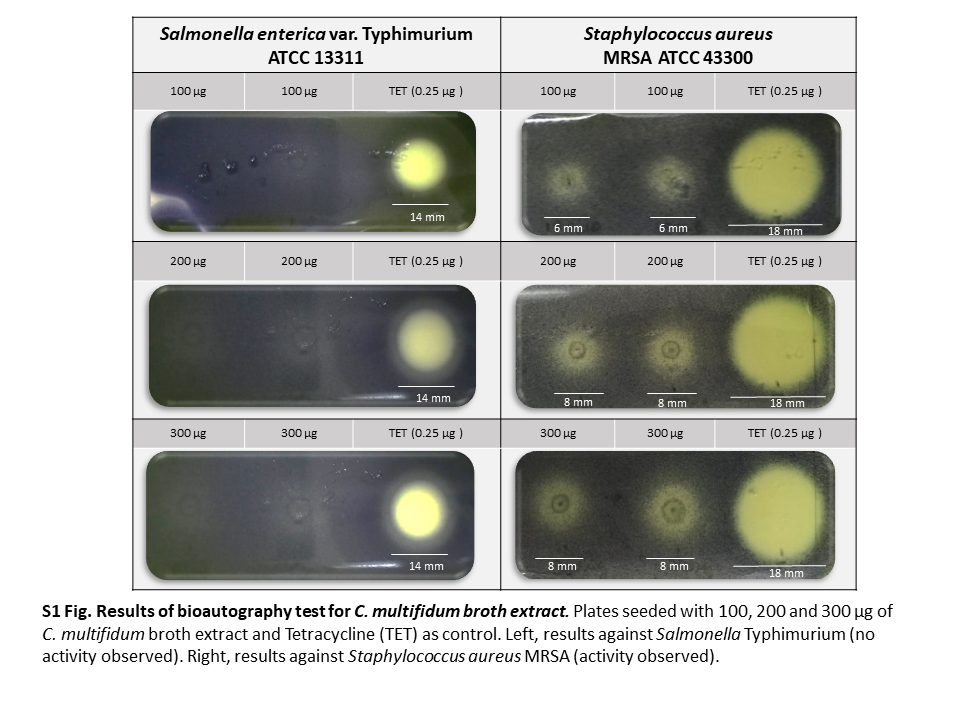

Supplement: S1 Fig — (TIF) [file pone.0218837.s002.tif]
