# Supplementary material for: Identification and characterization of compounds from Chrysosporium multifidum, a fungus with moderate antimicrobial activity isolated from Hermetia illucens gut microbiota
Source: PLoS One. 2019 Dec 20;14(12):e0218837. doi: 10.1371/journal.pone.0218837 (PMC6924690; doi:10.1371/journal.pone.0218837)
Supplement: S1 Data — (DOCX) [file pone.0218837.s004.docx]

**S2 Fig 1.** ESI-MS in positive mode of compound **1**

**S2 Fig 2.** ^1^H NMR (500 MHz, CDCl_3_) spectrum of compound **1**

**S2 Fig 3.** ^13^C NMR (125 MHz, CDCl_3_) spectrum of compound **1**

**S2 Fig 4.** ESI-MS in positive mode of compound **2**

**S2 Fig 5.** ^1^H NMR (300 MHz, CDCl_3_) spectrum of compound **2**

**S2 Fig 6.** ^13^C NMR (75 MHz, CDCl_3_) spectrum of compound **2**

**S2 Fig 7.** ESI-MS in positive mode of compound **3**

**S2 Fig 8.** ^1^H NMR (300 MHz, CDCl_3_) spectrum of compound **3**

**S2 Fig 9.** ^13^C NMR (75 MHz, CDCl_3_) spectrum of compound **3**

**S2 Fig 10.** ESI-MS in positive mode of compound **4**

**S2 Fig 11.** ^1^H NMR (500 MHz, CDCl_3_) spectrum of compound **4**

**S2 Fig 12.** ^13^C NMR (125 MHz, CDCl_3_) spectrum of compound **4**

**S2 Fig 13.** ESI-MS in positive mode of compound **5**

**S2 Fig 14.** ^1^H NMR (300 MHz, CDCl_3_) spectrum of compound **5**

**S2 Fig 15.** ^13^C NMR (75 MHz, CDCl_3_) spectrum of compound **5**

**S2 Fig 16.** ESI-MS in positive mode of compound **6**

**S2 Fig 17.** ^1^H NMR (500 MHz, MeOD) spectrum of compound **6**

**S2 Fig 18.** ^13^C NMR (125 MHz, MeOD) spectrum of compound **6**

**S2 Fig 19.** ESI-MS in positive mode of compound **7**

**S2 Fig 20.** ^1^H NMR (300 MHz, MeOD) spectrum of compound **7**

**S2 Fig 21.** ^13^C NMR (75 MHz, MeOD) spectrum of compound **7**
