# Supplementary material for: Identification and characterization of compounds from Chrysosporium multifidum, a fungus with moderate antimicrobial activity isolated from Hermetia illucens gut microbiota
Source: PLoS One. 2019 Dec 20;14(12):e0218837. doi: 10.1371/journal.pone.0218837 (PMC6924690; doi:10.1371/journal.pone.0218837)
Supplement: S2 Data — (DOCX) [file pone.0218837.s005.docx]

***Spectroscopic data for compounds 1 to 7***

4-methoxy-2H-pyran-2-one (**1**): white amorphous solid; ^l^H-NMR (CDC1_3_): 3.81 (s, 3H, H-1’), 5.54 (d, J = 0.8, 1H, H-3), 6.01 (dd, J = 5.9, 2.4, 1H, H-5), 7.35 (dd, J=5.8, 0.7, 1H, H-6); ^l3^C-NMR (CDC1_3_): 56.1 (C-1’), 90.6 (C-3), 103.6 (C-5), 151.4 (C-6), 164.2 (C-4), 170.4 (C-2); HRESIMS m/z 127.0388 [M+H]^+^ (calculated for C_6_H_7_O_3_, 127.0395).

4-methoxy-6-pentyl-2H-pyran-2-one (**2**): white amorphous solid; ^l^H-NMR (CDC1_3_): 0.89 (t, J = 6.8 Hz, 3H, H-5’), 1.23–1.37 (m, 4H, H-3’, H-4’), 1.59–1.70 (m, 2H, H-2’), 2.43 (t, J = 7.6 Hz, 2H, H-1’), 3.79 (s, 3H, H-6’), 5.40 (d, J = 2.3 Hz, 1H, H-3), 5.76 (dd, J = 2.4 Hz, 0.9, 1H, H-5); ^l3^C-NMR (CDC1_3_): 13.9 (C-5’), 22.3 (C-4’), 26.3 (C-3’), 31.1 (C-2’), 33.6 (C-1’), 55.8 (C-6’), 87.5 (C-3), 99.6 (C-5), 165.1 (C-4), 165.8 (C-6), 171.3 (C-2); HRESIMS m/z 197.1173 [M+H]^+^ (calculated for C_11_H_17_O_3_, 197.1178).

6-(1-hydroxypentyl)-4-methoxy-pyran-2-one (**3**): yellow oil, ^l^H-NMR (CDC1_3_): 0.95–0.84 (m, 3H, H-5’), 1.24–1.47 (m, 4H, H-3’, H-4’), 1.61–1.91 (m, 2H, H-2’), 4.37 (ddd, J = 8.0, 4.7, 0.9 Hz, 1H, H-1’), 3.79 (s, 3H, H-6’), 5.41 (d, J = 2.3 Hz, 1H, H-3), 6.08 (dd, J = 2.3, 0.8 Hz, 1H, H-5); ^l3^C-NMR (CDC1_3_): 13.9 (C-5’), 22.4 (C-4’), 27.2 (C-3’), 34.9 (C-2’), 70.07 (C-1’), 56.0 (C-6’), 88.1 (C-3), 98.6 (C-5), 164.5 (C-4), 166.5 (C-6), 171.4 (C-2); HRESIMS m/z 213.1124 [M+H]^+^ (calculated for C_11_H_17_O_4_, 213.1127).

6-[8-propyloxiran-1-yl]-4-methoxy-pyran-2-one (**4**): yellow oil, ^l^H-NMR (MeOD):1.02 (t, J = 7.3 Hz, 3H, H-5’), 1.53–1.72 (m, 4H, H-3’, H-4’), 3.27 (ddd, J = 6.2, 5.0, 2.0 Hz, 1H, H-2’), 3.52 (d, J = 2.0 Hz, 1H, H-1’), 3.89 (s, 3H, H-6’), 5.63 (d, J = 2.3 Hz, 1H, H-3), 6.26 (d, J = 2.3 Hz, 1H, H-5); ^l3^C-NMR (MeOD): 14.6 (C-5’), 20.1 (C-4’), 34.7 (C-3’), 55.1 (C-1’), 57.5 (C-6’), 63.1 (C-2’), 89.7 (C-3), 102.5 (C-5), 161.9 (C-6), 166.3 (C-2), 173.2 (C-4); HRESIMS m/z 211.0962 [M+H]^+^ (calculated for C_11_H_15_O_4_, 211.0970).

pestalotin (**5**): brown oil, ^l^H-NMR (CDC1_3_): 0.91 (t, J = 7.2 Hz, 3H, H-5’), 1.35 – 1.64 (m, 6H, H-2’, H-3’, H-4’), 2.24 (dd, J = 17.1, 3.8 Hz, 1H, H-5a), 2.79 (ddd, J = 17.1, 13.0, 1.8 Hz, 1H, H-5b), 3.62 (dt, J = 8.7, 4.4 Hz, 1H, H-1’), 3.76 (s, 3H, H-6’), 4.29 (ddd, J = 12.9, 4.4, 3.8 Hz, 1H, H-6), 5.14 (d, J = 1.8 Hz, 1H, H-3); ^l3^C-NMR (CDC1_3_): 14.1 (C-5’), 22.7 (C-4’), 27.7 (C-3’), 29.8 (C-5), 32.5 (C-2’), 56.3 (C-6’), 90.2 (C-3), 166.9 (C-4), 173.3 (C-2); HRESIMS m/z 215.1279 [M+H]^+^ (calculated for C_11_H_19_O_4_, 215.1283).

5,6-dihydro-4-methoxy-6-(pentanoyloxy)-2H-pyran-2-one (**6**): brown oil, ^l^H-NMR (MeOD): 0.94 (t, J = 7.4 Hz, 3H, H-5’), 1.36 (dq, J = 14.7, 7.4 Hz, 2H, H-4’), 1.58 (dt, J = 14.8, 7.4 Hz, 2H, H-3’), 2.60 – 2.76 (m, 2H, H-2’), 2.83 (dd, J = 6.8, 0.7 Hz, 2H, H-5), 3.81 (s, 3H, H-6’), 5.00 (t, J = 6.7 Hz, 1H, H-6), 5.23 (s, 1H, H-3).; ^l3^C-NMR (MeOD): 14.2 (C-5’), 23.2 (C-4’), 26.3 (C-3’), 29.4 (C-5), 39.12 (C-2’), 57.1 (C-6’), 80.3 (C-6), 90.7 (C-3), 168.4 (C-2), 174.8 (C-4), 208.0 (C-1’); HRESIMS m/z 213.1122 [M+H]^+^ (calculated for C_11_H_17_O_4_, 213.1127).

cyclo-(L-Pro-L-Phe) (**7**): colorless amorphous solid, ^l^H-NMR (MeOD): 0.95 (d, J = 6.9 Hz, 3H, H-11), 1.11 (d, J = 7.3 Hz, 3H, H-12), 1.87–2.09 (m, 3H, H-4, H-5a ), 2.25 – 2.41 (m, 1H, H-5b), 2.45-2.56 (m, 1H, H-10), 3.44 – 3.66 (m, 2H, H-3), 4.04-4.06 (m, 1H, H-9), 4.22 (m, 1H, H-6); ^l3^C-NMR (MeOD): 16.7 (C-11), 18.9 (C-12), 23.3 (C-4), 29.5 (C-5), 29.9 (C-10), 46.2 (C-3), 60.0 (C-6), 61.5 (C-9), 167.6 (C-1), 172.6 (C-7); HRESIMS m/z 197.1283 [M+H]^+^ (calculated for C_10_H_17_N_2_O_2_, 197.1290).
